# The effect of botulinum toxin injection dose on the appearance of surgical scar

**DOI:** 10.1038/s41598-021-93203-x

**Published:** 2021-07-01

**Authors:** Zhiyou Chen, Zong Chen, Ran Pang, Zhiru Wei, Han Zhang, Wenhui Liu, Guangshuai Li

**Affiliations:** Department of Plastic and Reconstructive Surgery, the First Affiliated Hospital of Zhengzhou University, No.1 Eastern Jianshe Road, Zhengzhou, 450052 China

**Keywords:** Clinical trial design, Outcomes research

## Abstract

Early postoperative injection of botulinum toxin type A (BTxA) can reduce surgical scar hypertrophy. BTxA injection at different time points is associated with different levels of efficacy, but the efficacy of different doses of BTxA for scar management has not investigated. The purpose of this study was to investigate the effect of different doses of BTxA administered early after surgery on scar improvement through a split-scar experiment. The study included 22 patients who underwent surgery between September 2019 and October 2020. High- and low-dose BTxA was randomly administered into each half of the surgical wound closure immediately after surgery. One half of the incision was injected with a low dose (4 U) of BTxA, and the other half was injected with a high dose (8 U). The scars were then evaluated at postoperative 6 months using the modified Stony Brook Scar Evaluation Scale (mSBSES), and patient satisfaction was evaluated using the Visual Analogue Scale (VAS). The occurrence of complications or adverse events was also recorded. Twenty patients completed the study and were analyzed. Compared with the low-dose sides, the high-dose sides had significantly better mSBSES scores and significantly higher VAS scores (*p* < 0.01, respectively). No serious adverse reactions or post-injection complications were observed. Immediately after the operation, high-dose BTxA (that is within the therapeutic range) injection improved the appearance of postoperative scar more than low-dose injection.

## Introduction

In patients who undergo invasive surgical procedures, the cosmetic scars that appear after wound healing can cause distress^1^. Multiple factors are associated with undesirable scars, including the surgical technique, anatomical regions, skin tension, postoperative infection, and immunologic responses^2^. Early treatment of surgical scars can result in better appearance and decrease the need for treatment in later stages^3^. Various treatments, such as compression therapy, radiation therapy, silicone gel therapy, and laser therapy, have proven to be helpful, but most of these therapies have been unsatisfactory^4^.

In the last ten years, several studies have indicated that botulinum toxin type A (BTxA) has a positive effect on the prevention and treatment of scars, and these include human studies too^5–8^. For example, Gassner et al. reported that BTxA can fix the underlying muscle tissue to reduce wound tension during scar formation^9^. Further, Xiao et al. confirmed that continuous injection of BTxA can reduce the thickness and amount of collagen deposition and decrease the degree of hypertrophic scarring^10^. A proven early treatment method to prevent scar formation is injection of BTxA into surgical lesions at different postoperative times^11^. In different studies, the injection time of BTxA has been reported from immediately after surgery to 9 days after surgery^12–14^. Hu et al. showed that BTxA may be more beneficial in the early stages of wound healing, and that injection of BTxA immediately after wound closure can provide excellent results for facial surgical scars^15^. However, the effects of different doses of BTxA has not yet been studied with a split-scar experiment. Therefore, this prospective, split-scar, randomized controlled trial was performed to investigate the effect of different doses of paralesional BTxA administration on scar cosmesis after surgical excision.

## Patients and methods

**Figure 1:**
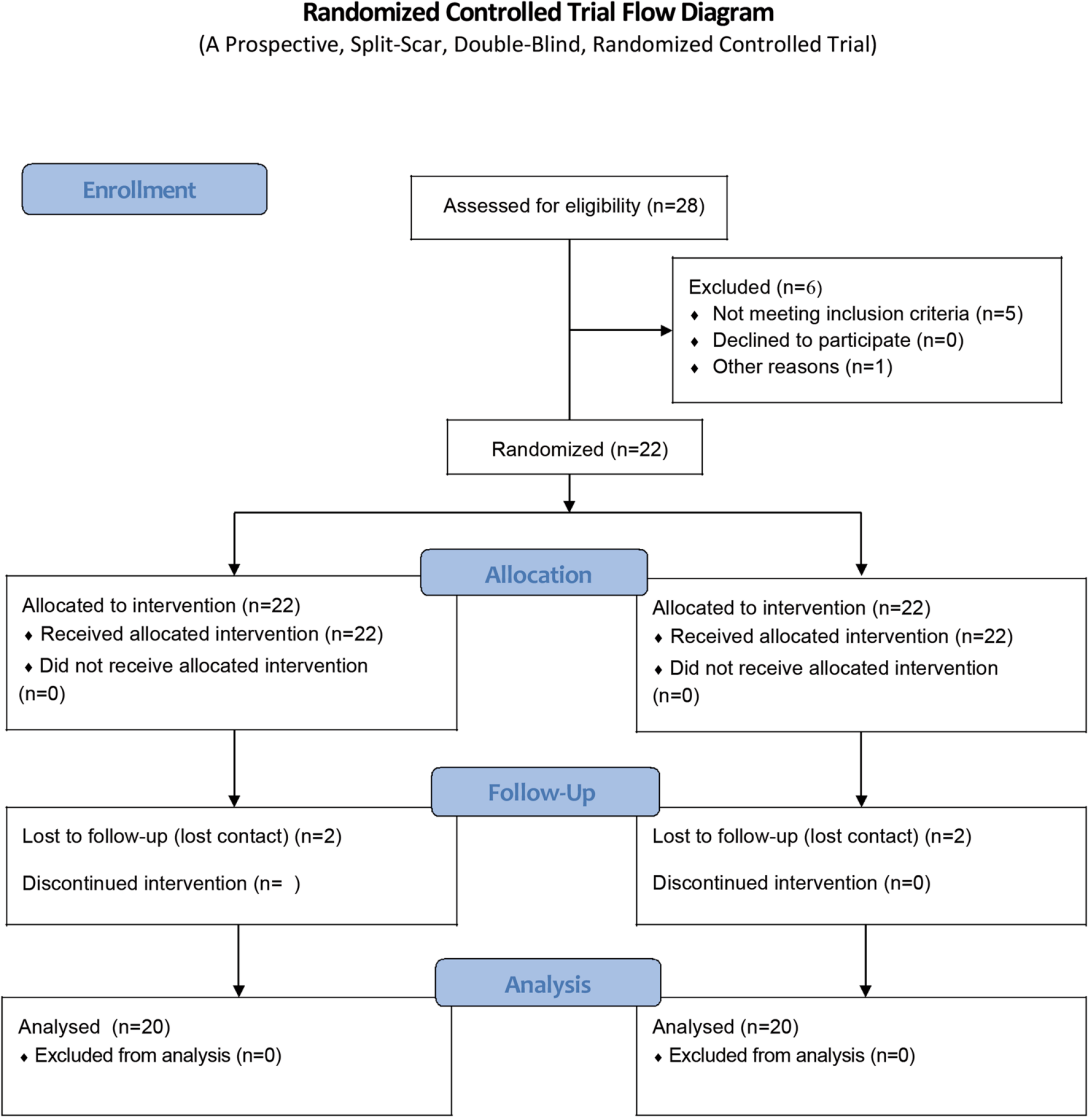
Randomized controlled trial flow diagram.

The present study was approved by the Institutional Review Board of the First Affiliated Hospital of Zhengzhou University. The local ethics committee of our hospital approved this study, which conformed to the provisions of the Declaration of Helsinki. This clinical trial has been registered on Chinese Clinical Trial Registry (ChiCTR, Registration date was 05/01/2021, Registration number was *ChiCTR2100041766*) (www.chictr.org.cn). All participants were informed about the study through a clear and simple written description of the procedure to ensure their understanding, and they provided their informed consent for participation. Randomized Controlled Trial flow diagram was shown in Fig. 1.

### Sample size

The sample size was calculated based on the method described in previously published literature in 2018^15^: An increase of 1 in the VAS score after treatment was considered as clinically significant^15^. Based on this criterion, approximately 18 wounds per group would be necessary to provide a result of real significance with the same standard deviation considering a standard type 1 α error of 0.05 and power of 0.8. Assuming a 10% noncompliance rate for follow-up (evaluation), the required sample size was determined as 20 patients.

The study included participants aged 18 years and above who were scheduled to undergo superficial mass excision (skin type of the patients: Fitzpatrick skin type III or IV) at the Department of Plastic Surgery, First Affiliated Hospital of Zhengzhou University, between September 2019 and October 2020.

The exclusion criteria were (1) known BTxA or albumin allergy, (2) BTxA injection within the past 6 months, (3) a history of neuromuscular disorders, (4) a history of hypertrophic scars and keloids, and (5) current pregnancy or breast feeding.

### Procedure

Z. C. generated the random allocation sequence, enrolled and assigned participants. A random number generator was used to generate ones and twos (each time a random number was generated, the maximum value was 2 and the minimum value was 1), which designated left and right, respectively, or upper and lower, respectively. Accordingly, half part of individual participant’s wound was randomly assigned to one of two treatment groups: the low-dose group received 4 U of BTxA and the high-dose group received 8 U at each administration. Vials containing 100 U of BTxA (Lanzhou Biochemical Company, Lanzhou City, People’s Republic of China) was diluted in 0.9% saline solution to achieve a concentration of 4 U per 0.1 mL or 8 U per 0.1 mL. The solutions were same in appearance. Use the same 1 mL syringe (29G needle) for the injection.

After tumor resection, incision tension was assessed. Vertical mattress suture was performed on the subcutaneous layer and dermis with 3–0 to 5–0 absorbable sutures, respectively. After closing the incision, discontinuous epidermal suture was performed with 6–0 or 7–0 nylon sutures. After skin closure, the wound was marked at the midline point. The postoperative wound was treated, with each side randomized to receive either a low dose of BTxA (4 U at every point, with an interval of 1 cm) or a high dose of BTxA (8 U at every point, with an interval of 1 cm) injected intradermally from a site 5 mm away from the wound edges. Patients, injector (Z.R. W.) and scar evaluators (R. P. and H. Z.) were blinded to the dose received.

After operation, the patient was observed in the recovery room for 30 min and the discomfort and adverse reactions (if any) were recorded. Disinfect and replace excipients every 2–3 days according to the surgical site and wound healing. The time of suture removal depends on the site of operation. No additional anti-scar therapy, such as stress therapy or silicone application, was given after surgery. The occurrence of complications or other discomfort was also recorded during the follow-up.

### Evaluation of clinical effect

The cohort was observed over a 6-month postoperative follow-up period. At each of the follow-up visits, two plastic surgeons (R. P. and H. Z.) assessed and scored the left and right sides of the incision independently using the mSBSES (The mSBSES contains four subcategories, as shown in Table 1) and VAS (from 0 to 10: 0 = worst and 10 = best) and took standardized digital photographs of the scars^14,15^. The differences in mSBSES score and VAS score between the two groups was used as the primary outcome, and the occurrence of complications or adverse events was considered as a secondary outcome^16^. The patients were required to report adverse events. Throughout the study period, the patients were blinded to the BTxA dose administered to each side.

**Table 1:** Modified Stony Brook scar evaluation scale (mSBSES).

Scar category	Score
**Width**	
Scar widening prominent, width > 2 mm	0
Scar widening present, width ≤ 2 mm	1
No scar widening	2
**Height**	
Prominent scar elevation	0
Scar elevation present	1
No scar elevation	2
**Color (redness)**	
Skin prominently more red than the surrounding skin	0
Scar more red than the surrounding skin	1
Scar of the same color or lighter than surrounding skin	2
**Incision line**	
Prominent incision line	0
Incision line present	1
Incision line absent	2

### Statistical analysis

IBM SPSS Version 22 (IBM Corp., Armonk, N.Y.) was used to analyze all data. Paired *t* tests were used to compare the summative scores for VAS, SBSES and its sub-items for each half of the scars. Non-parametric rank sum test was used for measurement data that did not conform to normal distribution (Shapiro–Wilk test was used for normality test, and Wilcoxon test was used for nonparametric test). Statistical significance was accepted at p < 0.05.

## Results

A total of 22 patients were enrolled. Twenty patients (11 male patients and 9 female patients) finally completed the entire study because two dropped out (loss of follow-up). The average patient age was 37 years (range, 18–52 years). A total of 40 wounds in 20 patients were analyzed based on the split-scar method. The surgical sites were: face in 5 cases, neck in 3 cases, upper extremity in 5 cases, chest wall in 1 cases, back in 3 cases and abdominal wall in 3 case. The original diagnoses included congenital melanocytic nevi (11/20, 55%), superficial scar (4/20, 20%), seborrheic keratosis (2/20, 10%), sebaceous nevus (2/20, 10%), and keratoacanthoma (1/20, 5%). The mean scar length was 8.64 cm (range, 5 to 15 cm), and the average amount of BTA injected was 80.40 U (range, 48 to 156 U). No complications were encountered.

### Primary outcome

When the scars were evaluated using the modified SBSES after 6 months by plastic surgeons, the mean score was found to be 5.90 ± 1.59 for the high-dose injection side and 4.15 ± 1.31 for the low-dose injection side (*p* < 0.01). Among the modified Stony Brook Scar Evaluation Scale subcategories, compared to the low-dose side, the high-dose side had significantly greater values for width (1.65 ± 0.49 versus 1.05 ± 0.69, *p* < 0.01) and incision visibility line (1.05 ± 0.51 versus 0.50 ± 0.61, *p* < 0.01). However, there was no significant difference in height (1.65 ± 0.59 versus 1.35 ± 0.59, *p* = 0.11) and color (redness) (1.55 ± 0.60 versus 1.25 ± 0.64, *p* = 0.13) (Table 2).

**Table 2:** Modified Stony Brook scar evaluation scale (mSBSES) of the high-dose and low-dose injection sides at 6 months after BTxA injection.

	High-dose injection side	Low-dose injection side	*p*
Width	1.65 ± 0.49	1.05 ± 0.69	< 0.01*
Height	1.65 ± 0.59	1.35 ± 0.59	0.11
Color (redness)	1.55 ± 0.60	1.25 ± 0.64	0.13
Incision line	1.05 ± 0.51	0.50 ± 0.61	< 0.01*
mSBSES	5.9 ± 1.59	4.15 ± 1.31	< 0.01*

*Significant different. Data is represented as mean ± standard deviation (M ± SD).

Patient satisfaction was evaluated using VAS. At the 6-month follow-up, higher satisfaction was reported for the high-dose injection side, and the mean VAS score for the high-dose side was 7.85 ± 1.27 and the low-dose side was 5.20 ± 1.40, with a statistically significant difference between the two sides (*p* < 0.01).

**Figure 2:**
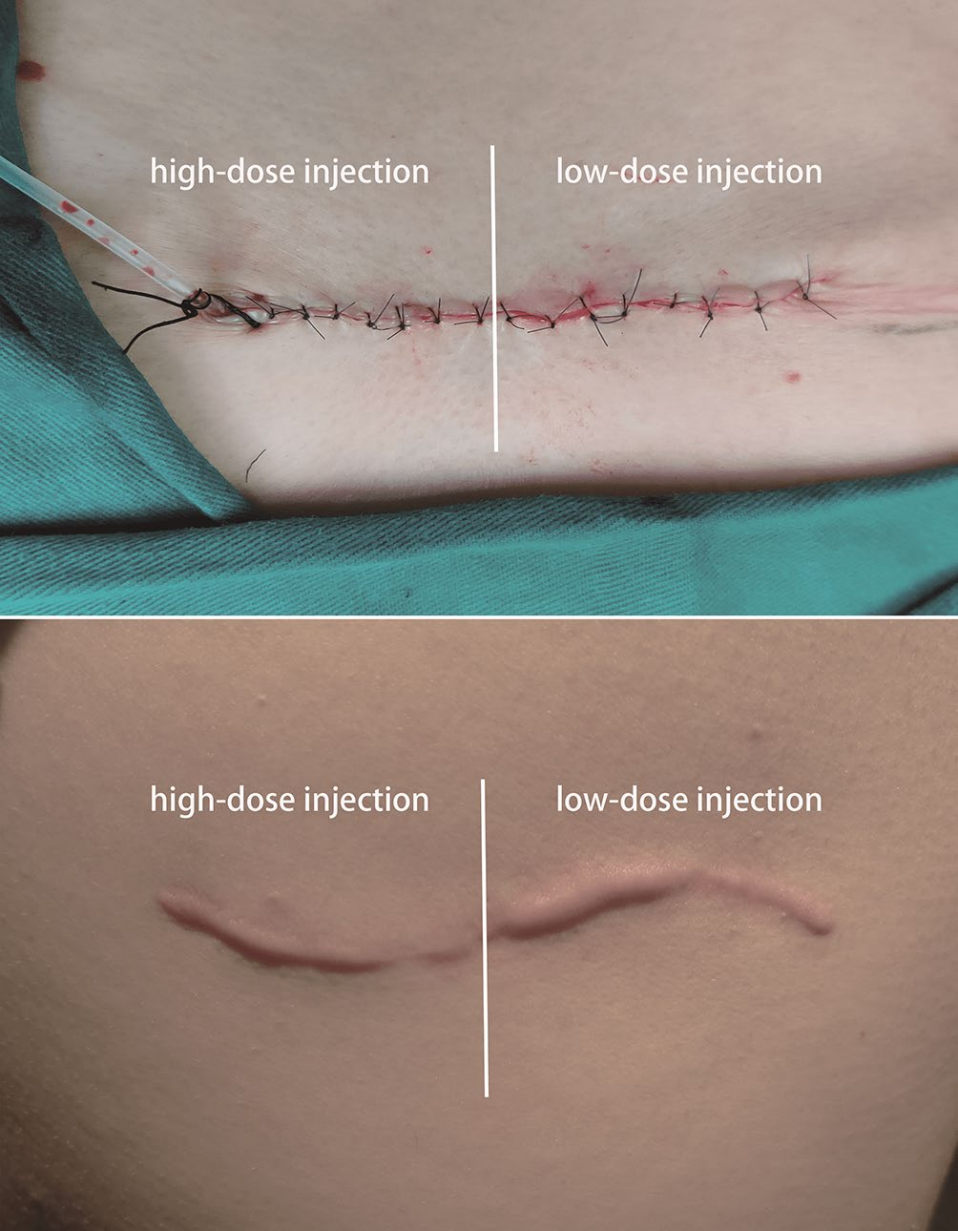
Right back scar at baseline (*above*) and at 6-month follow-up (*below*). The right half of the scar was treated with high-dose botulinum toxin type A (BTxA) and the left half was treated with low-dose BTxA.

**Figure 3:**
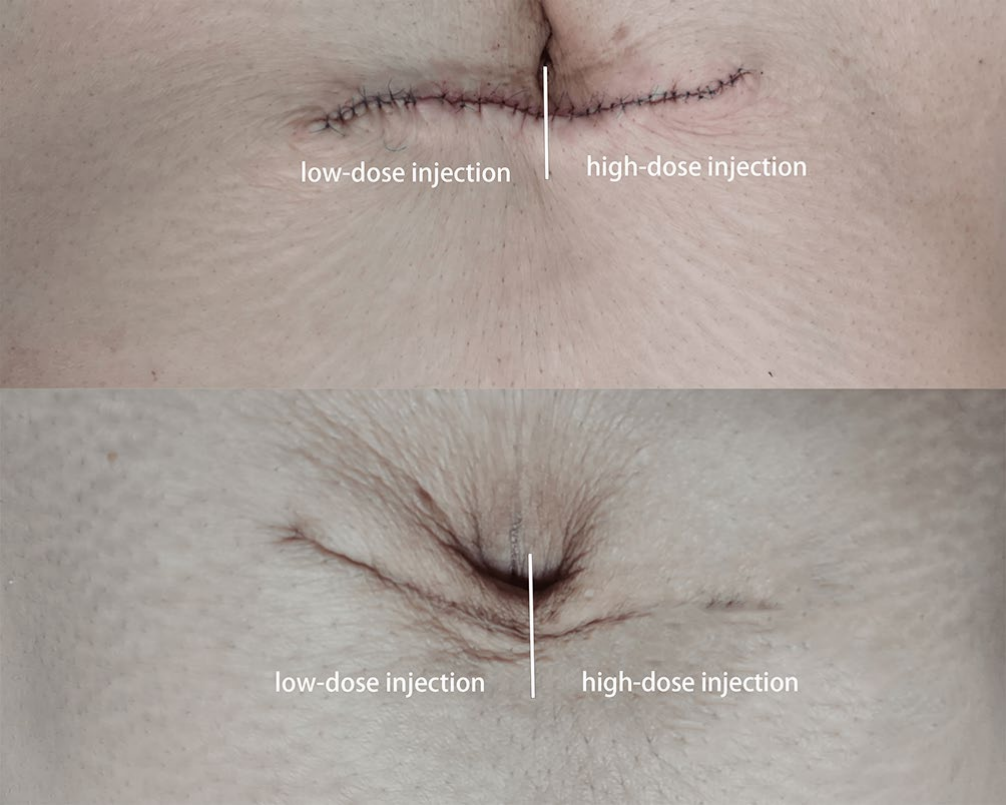
Abdominal scar at baseline (*above*) and at 6-month follow-up (*below*). The right half of the scar was treated with high-dose botulinum toxin type A (BTxA) and the left half was treated with low-dose BTxA.

Photographs of representative patients are shown in Figs. 2 and 3.

### Secondary outcome

No serious adverse reactions or post-injection complications were observed (such as hematoma, infection, botulism, allergies, or muscle paralysis).

## Discussion

The present split-scar study explores the impact of immediate postoperative administration of high and low doses of BTxA around the area of incision on postoperative scar prevention. In our study, the high-dose and low-dose injection sides showed significant differences, with the high BTxA dose exhibiting better results. With regard to the modified SBSES subcategories, height and color (redness) were not significantly different between the two sides, but the high-dose injection had significantly better results in terms of the other two subcategories, namely, width and incision visibility line. In contrast, Kim et al. used a split-scar study to compare BTxA and non-BTxA injection sides and observed a significant effect in all four modified SBSES subcategories^14^. This difference could be explained by the small sample size of our study.

What needs illustration is that the tumors of the patients in this study generally did not invade the muscle, so the muscle tissue remained intact after resection (no muscular tension). After intradermal injection of botulinum toxin, the high-dose group showed better scar appearance. This suggests that the role of botulinum toxin in preventing scar formation is more dependent on non-neuromuscular pathways than on the relaxation of muscle. A series of studies by Xiao et al^10,17–19^ systematically elucidate the main mechanisms of botulinum toxin preventing scar formation: (i) Botulinum toxin can inhibit the proliferation of fibroblasts, promote their apoptosis, and inhibit their differentiation into myofibroblasts; (ii) Botulinum toxin can inhibit the expression and secretion of fibrosis related factors. (iii) At the same time, botulinum toxin can change collagen deposition and induce collagen remodeling to some extent while reducing tension. To be specific, Botulinum toxin type A can work as a downstream regulator of TGF-β1 secreted by macrophages, which can reduce the expression of connective tissue growth factor. And botulinum toxin type A acts on the TGF-β1/Smad pathway at the molecular level, thereby inhibiting Scars formed by fibroblasts^11,18^.

Gassner et al. described a mean dose of 30 U for 2- to 4-cm forehead wounds in a 2006 report^12^. The reported dose varies from 2.5 to 10 U for each 1-cm scar in different studies^12,20^ and most studies use a dose of 5 U for each 1-cm scar to investigate the effect of BTxA on postoperative scar management^4,21^. Therefore, in this study, 4 U was used as the low dose and 8 U as the high dose for each 1-cm scar. Additionally, the wounds were divided into high- and low-dose sides, and this greatly reduced the interference of unrelated confounding factors. The high-dose botulinum toxin was found to be superior to the low-dose toxin in terms of reducing the tension around the incision and inhibiting scar hypertrophy. In agreement with these findings, clear differences have been reported in the effects of different doses of BTxA on crow’s feet, primary palmar hyperhidrosis, and gummy smile^22–25^.

The difference in the effect of different doses of BTxA may be related to differences in the diffusion rate of various doses^26^. A study reported that a gradient of denervation occurred throughout the entire muscle with no apparent endpoint when BTxA was administered at doses of 5–10 IU, and both the magnitude of denervation and the extent of the gradient were dose dependent^27^. Therefore, different doses of BTxA may have different effects on the muscles at the injection site. The results of a dose-ranging, electroneurographic study investigating the dose equivalence and diffusion characteristics of BTxA in 2008 showed significant and similar reductions in compound muscle action potential amplitude in the extensor digitorum brevis 2 weeks after injection, with the effects lasting for 12 weeks. Further, the reduction in amplitude increased with increasing doses and with increasing concentration^27,28^. Thus, the dose-dependent effects observed in this study could be explained by these muscle-related mechanisms of BTxA. According to these findings, many other studies have also shown that for botulinum toxin, its volume, dose, and accuracy have the greatest impact on clinical outcomes^29–31^.

The main limitation of our study is the small sample size. Therefore, studies with a larger sample size are necessary to confirm the results. Second, it will be important to perform studies to investigate a range of BTxA doses within the therapeutic dose in order to determine whether the BTxA dose is directly proportional to the effectiveness of scar prevention.

## Conclusion

In the present study, the results show that the high dose of BTxA was more effective than the low dose in the management of scar hypertrophy. These findings indicate that early postoperative high-dose BTxA injections can provide better cosmetic effects than low-dose injections. Therefore, we recommend a high-dose BTxA injection immediately after the procedure to achieve a better scar beautification effect.
